# Assessment of Function in Patients after Calcaneal Fracture Treatment with the Ilizarov Method

**DOI:** 10.3390/jcm13164671

**Published:** 2024-08-09

**Authors:** Marcin Pelc, Władysław Hryniuk, Andrzej Bobiński, Joanna Kochańska-Bieri, Łukasz Tomczyk, Daniele Pili, Wiktor Urbański, Marcin Lech, Piotr Morasiewicz

**Affiliations:** 1 Institute of Medical Sciences, Opole University, 45-401 Opole, Poland; 2 Department of Orthopaedic and Trauma Surgery, Institute of Medical Sciences, University of Opole, 45-401 Opole, Poland; 3 Universitätsspital CH, University of Basel, 4031 Basel, Switzerland; 4 Department of Food Safety and Quality Management, Poznan University of Life Sciences, 60-637 Poznan, Poland; 5 Orthopedic and Trauma Department, G B. Mangioni Hospital, 23900 Lecco, Italy; 6 Department of Neurosurgery, Wroclaw Medical University, 50-556 Wroclaw, Poland; 7 Department of Orthopaedics, Traumatology and Hand Surgery, Wroclaw Medical University, 50-556 Wroclaw, Poland

**Keywords:** calcaneal fracture, Ilizarov method, functional assessment, physical activity, range of motion

## Abstract

**Background:** Up to 75% of calcaneal fractures are intra-articular fractures, which may severely impair foot function and lead to disability. **Methods:** We retrospectively analyzed 21 patients with intra-articular calcaneal fractures who had been treated with the Ilizarov method in the period 2021–2022. The mean patient age was 47 years (range 25–67 years). We analyzed the following functional parameters: foot function with a revised foot function index (FFI-R) questionnaire and the level of physical activity, with the University of California Los Angeles (UCLA) activity scale, a visual analog scale (VAS), and a Grimby physical activity level scale; and ankle range of motion. **Results:** We observed a significant improvement in the UCLA activity scores and Grimby activity score at long-term follow-up. Functional outcomes based on the FFI-R questionnaires showed an improvement, from 292 points prior to surgery to 127 points at follow-up, *p* = 0.013. The post-treatment follow-up measurements revealed a median dorsiflexion at the treated ankle joint of 20 degrees, whereas that at the intact ankle was 40 degrees, *p* = 0.007. The plantar flexion showed asymmetry, with a median 15 degrees at the treated ankle and 30 degrees at the intact ankle, *p* = 0.007. The median range of inversion at the ankle joint was 5 degrees in the treated limb and 15 degrees in the intact limb, *p* = 0.039. **Conclusions:** Patients with calcaneal fractures treated with the Ilizarov method are recommended to have a longer and more intensive rehabilitation. The range of ankle motion in the treated limb was limited in comparison with that in the intact limb; however, this did not greatly affect the patients’ return to their earlier, pre-injury level of physical activity.

## 1. Introduction

Calcaneal fractures are among the most common tarsal fractures and usually result from high-energy trauma, such as road traffic accidents or falls from heights [1,2,3,4]. Up to 75% of calcaneal fractures are intra-articular fractures, which may severely impair foot function and lead to disability [2,4,5,6,7]. Regaining full functionality often requires a long time, and a number of patients struggle with reaching pre-injury levels of activity [2,3,4,7,8]. Consequently, calcaneal fractures have a considerable socioeconomic impact, since they mostly affect the working-age population [4,7,9]. Walking begins with placing the foot on the heel, which is why effective treatment of calcaneal fractures is so important and crucial [5,8]. Unfortunately, despite the development of techniques for internal and external stabilization of calcaneal fractures, there are no ideal methods of treating calcaneal fractures. Treatment of calcaneal fractures is often still difficult for surgeons and does not always provide good treatment results [2,3,4,5,6,7,10,11,12,13,14,15,16,17,18,19,20,21,22,23,24]. Treatment methods for intra-articular calcaneal fractures are a subject of ongoing discussions [3,10,11,12,13]. Some experts recommend open reduction and internal fixation [4,6,7,14], while others prefer the use of external fixators [2,3,5,8,10,11,12].

Despite advances in surgical techniques involving open reduction and internal fixation via lateral access, the functional outcomes are unsatisfactory in many cases [7,11,13].

The results from some studies on surgical techniques suggest that minimally invasive approaches and percutaneous reduction and stabilization methods are superior to traditional open reduction methods. In particular, the use of minimally invasive techniques is associated with a lower risk of complications related to soft tissue damage, resulting in shorter recovery times and better overall treatment outcomes [24]. Open reduction and internal fixation of calcaneal fractures are associated with a high risk of complications, reaching up to 33% [6,11,12,13]. The most common complications in open reduction and internal stabilization of calcaneal fractures include infections, delayed wound healing, soft tissue necrosis, and destabilization of the implants [6,11,12,13]. Another technique used in calcaneal fracture treatment is the Ilizarov method [5,6,8,10,11,12,13,15,16]. This involves external fixation of bone fragments and the restoration of calcaneal bone shape and foot structure [5,8,11,12,13]. Achieving the correct shape and three-dimensional structure of the calcaneus is one factor that determines good clinical outcomes and normal foot function in patients following surgical treatment [4,5,6,11,12,13]. Treatment of calcaneal fractures using external fixators, including the Ilizarov method, has an advantage over internal fixation with plates in the form of a lower incidence of complications, which is related to a less extensive surgical access [5,6,8,10,11,12,13,15,16]. The functional limitations observed after calcaneal fractures have been attributed to limited mobility, stiffness, and pain at the ankle joint [4,5,11]. However, they might be due to an altered geometric morphometry of the calcaneus and an uneven articular surface (posterior facet) [4,5,7,17]. Therefore, it is important to monitor ankle joint mobility and function parameters after calcaneal fracture treatment, starting from early stages of rehabilitation. This approach allows for therapeutic process adjustments, helps patients regain full functionality more rapidly, and reduces the risk of complications [17]. Previous Ilizarov treatment techniques for calcaneal fractures described the use of at least three Kirschner wires to stabilize the foot [10,11,12,13,15]. The greater the number of implants placed in the foot, the greater the risk of complications, including infection, swelling, and delayed wound healing [5,8,11]. The Polish modification of the Ilizarov method in the treatment of calcaneal fractures allows the foot to be stabilized with only one Kirschner wire [5,8]. Theoretically, a smaller number of implants placed in the foot during the treatment of calcaneal fractures should be associated with fewer complications and allow for better functional results of treatment [5,8].

Pelc et al., who assessed balance and weight distribution in the lower limbs following calcaneal fracture treatment with the Ilizarov method, reported normalized weight distribution and no differences between the patient group and the control group in the mean center of gravity sway area [8]. It was only the mean displacement of the center of gravity that was greater in the patient group than in the controls [8]. The available literature contains no studies assessing ankle functionality, activity levels, and the range of motion following calcaneal fracture treatment with the Ilizarov method. There have been only a handful of studies evaluating the range of motion at the ankle after calcaneal fracture treatment [18,19,20]; however, those patients had not been treated with the Ilizarov method. One study assessed the ankle functional capacity in patients after calcaneal fracture treatment with internal fixation [14].

We posed the hypothesis that the use of the Ilizarov method in treating calcaneal fractures will help improve functional parameters and the range of motion at the ankle joint and the levels of physical activity.

The purpose of our study was to assess functional outcomes after calcaneal fracture treatment with a Polish modification of the Ilizarov method.

## 2. Materials and Methods

**Figure 1:**
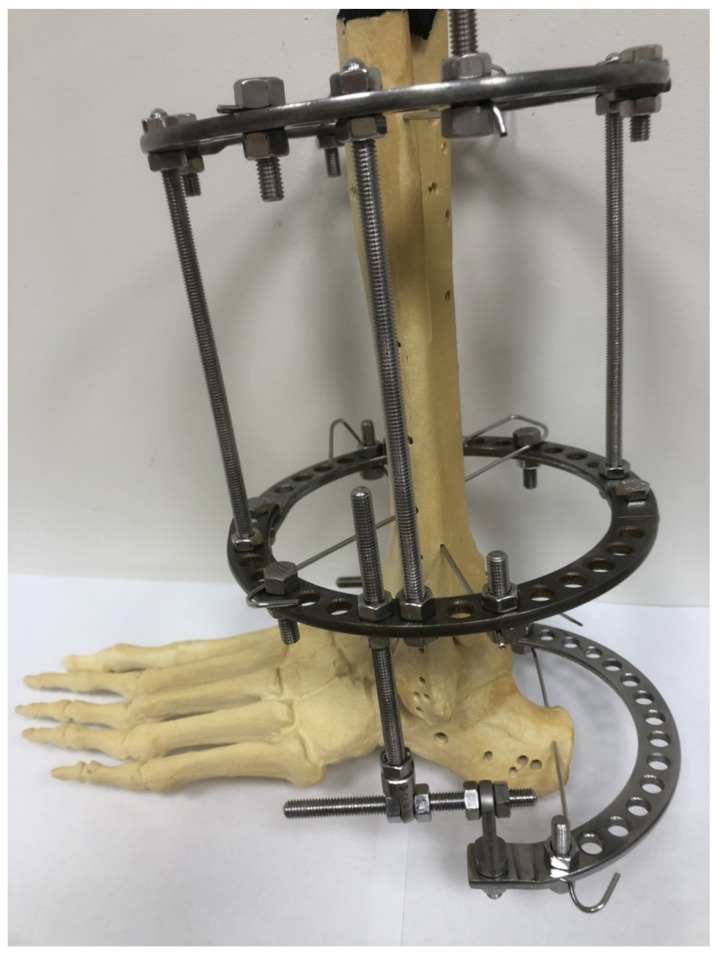
Model of the Polish modification of the Ilizarov external fixator in the treatment of calcaneal fractures.

This study was retrospective in nature. We analyzed patients with intra-articular calcaneal fractures who had been treated (at one center) with the use of a Polish modification of the Ilizarov method (Figure 1) in the period 2021–2022. Study inclusion criteria were a minimum follow-up of 2 years after treatment completion, intra-articular calcaneal fracture treated with a Polish modification of the Ilizarov method by the same orthopedic surgeon, complete medical records and data from functional assessment questionnaires (physical activity questionnaire, range of motion at the ankle joint), a lack of lower limb comorbidities, and informed consent to voluntarily participate in this study, with the option of withdrawing from the study at any time.

Those individuals who did not meet the above criteria were excluded from the study. This study was approved by the local ethics committee (Approval No. UO/0023/KB/2023). The study was conducted in accordance with the Declaration of Helsinki. All patients participating in the study gave voluntary and informed consent. All patients were informed about the voluntary nature of the study and about the possibility of resigning from participation in the study at any stage.

All patients underwent diagnostic imaging in the form of X-rays of the foot in AP and lateral projections, an axial image of the calcaneus and computed tomography of the foot and ankle joint. Patients in the emergency department were treated immediately after injury in a short leg cast. Stabilization of a calcaneal fracture using the Ilizarov method was performed by an experienced orthopedist. The operation was performed within 3 to 5 days of the fracture, depending on the availability of the operating room and the presence of the operator at work. Surgical treatment of calcaneal fractures was conducted with the use of a Polish modification of the Ilizarov external fixation method [5,8], shown in Figure 1. The external fixator was composed of two rings (secured to crural bones with Kirschner wires) and one half ring secured to the calcaneus with a Kirschner wire. The first Kirschner wire was inserted under fluoroscopy into the most proximal and dorsal calcaneal bone fragment. Subsequently, the half ring fixated to the calcaneus was secured to the distal leg ring via two connectors (composed of two perpendicular, threaded rods). Once the fixator was mounted onto the foot and leg, the calcaneal fracture was reduced under fluoroscopy. The Polish modification of the Ilizarov external fixator enables stabilization and a reduction in calcaneal fracture using only one Kirschner wire inserted into the calcaneus [5,8]. Thanks to the Polish modification of the Ilizarov external fixator, it is possible to stretch and correct the position of bone fragments in the frontal and sagittal planes [5,8]. The Polish modification of the Ilizarov external fixator design also enables the correction of valgus or varus deformation of the calcaneus [5,8]. The Ilizarov external fixator design we use also enables arthrodiastasis of the ankle joint and talocalcaneal joint, which is beneficial in the treatment process [5,11,13]. Postoperative rehabilitation was conducted according to a single protocol. On day one after surgery, the patients began walking with the use of two elbow crutches while bearing partial weight on the treated limb. Weight bearing was gradually increased, to the extent allowed by the patient’s pain tolerance. Pre-scheduled follow-up visits were to assess the progress of treatment. If the wounds healed well, patients were discharged home from the hospital ward on the first day after surgery. Follow-up X-rays were taken on the day of the procedure, two and six weeks after the procedure, and then every four weeks until bone union was achieved. When clinical and radiological evidence of union was observed, the fixator was loosened and the patient was allowed to walk with full weight bearing. The fixator was removed 7 days after it was loosened when a follow-up X-ray film showed no bone fragment displacement.

Study inclusion criteria restricted the study group to 21 patients, including 7 women and 14 men, who were treated for intra-articular calcaneal fracture. The Sanders classification-based fracture types in our patients were type 2 in three cases, type 3 in five cases, and type 4 in 13 cases. The mean patient age was 47 years (range 25–67 years), the mean body weight was 81 kg (range 61–130 kg), the mean height was 171 cm (range 152–188 cm), and the mean body mass index was 28 (range 24–40).

**Figure 2:**
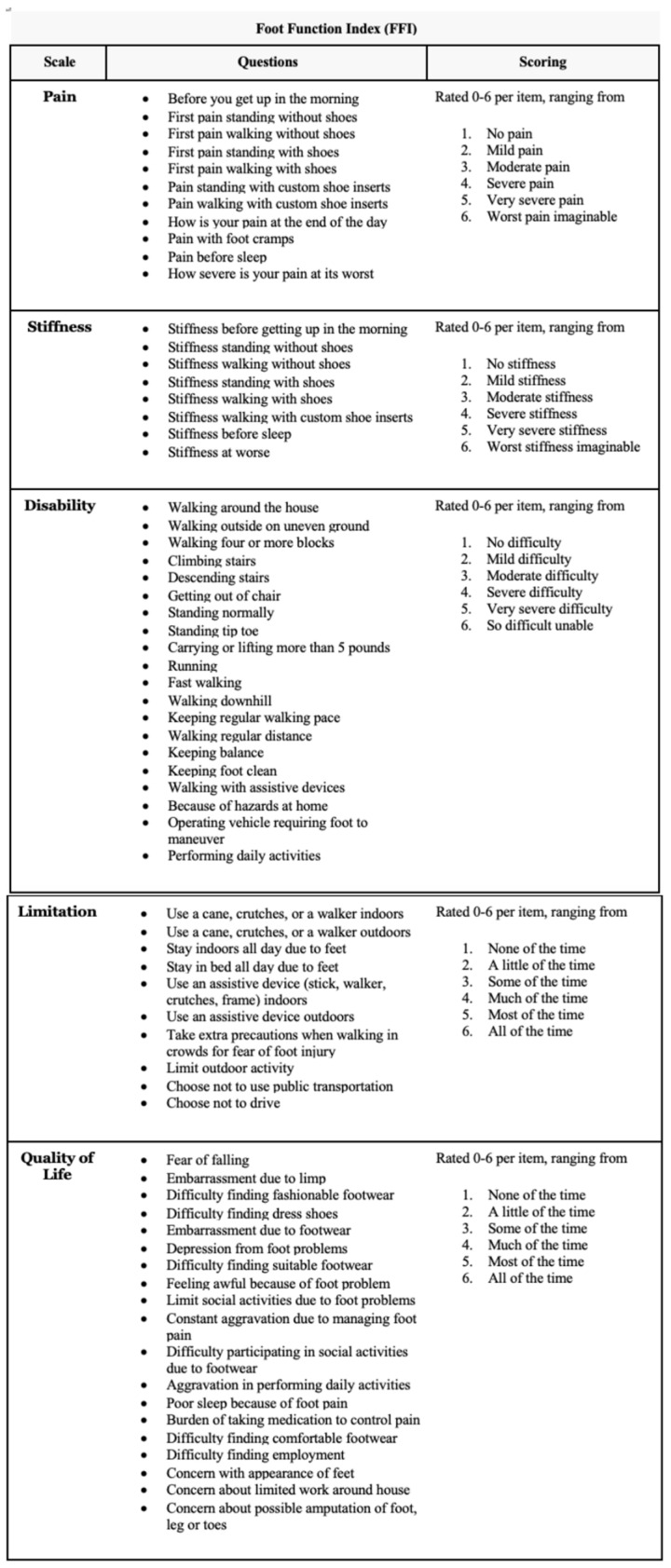
Foot function index-revised (FFI-R) questionnaire.

**Figure 3:**
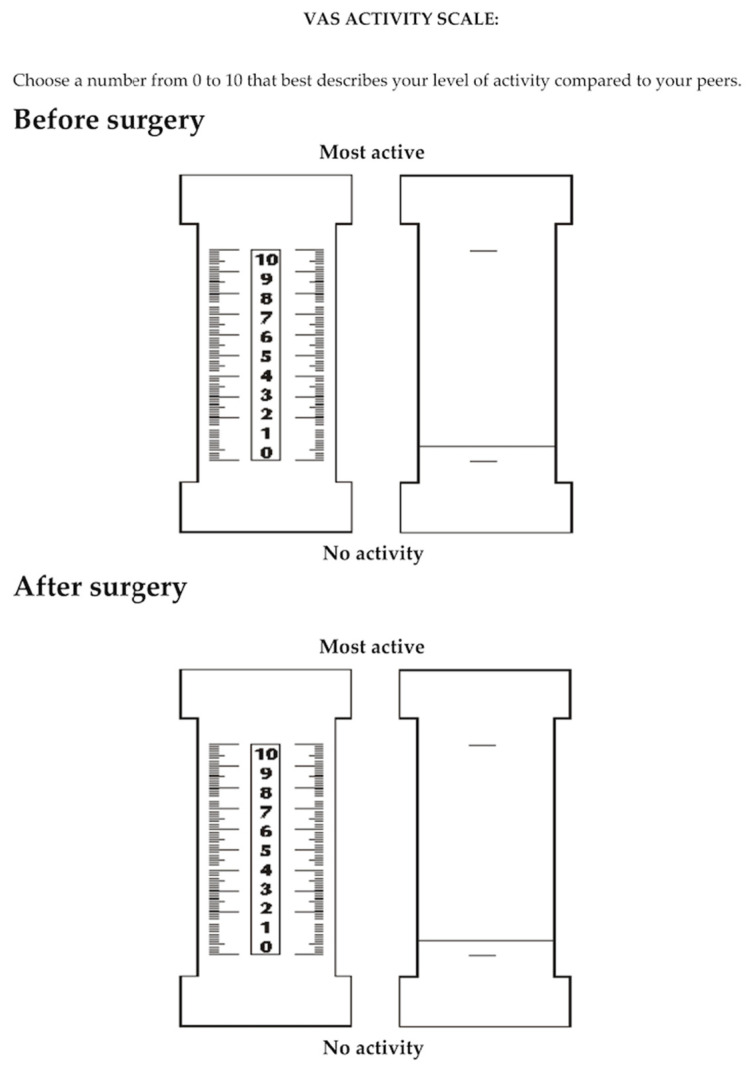
Ten-point activity visual analog scale (VAS).

In our study, we analyzed the following functional parameters: foot function with a revised foot function index (FFI-R) questionnaire [25,26] (Figure 2) and the level of physical activity, with a 10-point University of California Los Angeles (UCLA) activity scale [27], a 10-point activity visual analog scale (VAS) [28] (Figure 3), and a 6-point Grimby physical activity level scale [29].

In the study, we used an extended form of the foot function index-revised (FFI-R) questionnaire [25,26], which was created to measure foot function. It contains five subscales and a total of 68 questions. These subscales include foot pain (11 items), stiffness (8 items), difficulty related to foot functioning (20 items), activity limitation (10 items), and social functioning (19 items). All items in the questionnaire were based on a 6-point response scale, which was modified accordingly for each; for example, for the pain subscale: 1 = no pain, 2 = mild pain, 3 = moderate pain, 4 = severe pain, 5 = very severe pain, 6 = worst pain imaginable. On this scale, zero means the best functional result of the foot, while the higher the score, the worse the foot function. Additionally, we used the UCLA activity scale to assess the level of physical activity [27]. The level of physical activity was assessed on a 10-point scale based on 10 descriptive levels of activity. Each level of this scale corresponded to specific types of physical activity, ranging from very low activity (e.g., a sedentary lifestyle) to very high activity (e.g., regular participation in sports requiring high physical exertion). In the questionnaire, participants were asked about their participation in various activities and physical activities, and the researcher assessed and assigned them to the appropriate level. The activity visual analog scale (VAS) for assessing physical activity allowed for a subjective assessment of one’s physical activity over a specific period of time [28]. This scale included two extreme points: a point of 0 was a complete lack of physical activity, which indicated the lack of any form of movement or exercise. In turn, point 10 represented the highest possible level of physical activity, which referred to intense, regular, and demanding physical exercise. Subjects were asked to place their activity level on this scale, marking the point that they felt best reflected their daily physical activity. For the Grimby scale [29], designed to self-assess physical activity levels, activity levels were classified based on responses to the question: “Choose the one answer that best describes your level of physical activity”. Patients were given six options that detailed different levels of physical activity: 1. Almost no physical activity, 2. Mostly sedentary lifestyle with occasional walking and gardening, 3. Light exercise, 4. Moderate exercise of less than 2 h per week, 5. Moderate exercise of at least 3 h a week, 6. Regular vigorous exercise. Before completing each questionnaire, all respondents were instructed in detail on how to complete them. The instructions were clear and precise to ensure that all questions were understood and the forms were completed correctly. While completing the questionnaires, respondents could ask for help or clarification at any time if they encountered any difficulties or ambiguities related to the content of the questionnaire. This ensured that the collected data were of a high quality and reliable.

We compared functional parameters before surgery and those obtained after long-term postoperative follow-up. Ankle range of motion was measured manually with a goniometer in the treated and healthy limb and included dorsiflexion, plantar flexion, inversion, and eversion, with the results expressed in degrees.

### Statistical Analysis

Data were statistically analyzed using Statistica 13.3. The Shapiro–Wilk test was used to check for normality of distribution. The Wilcoxon signed-rank test was used to compare quantitative variables. The significance level was set at *p* < 0.05.

## 3. Results

We observed a significant improvement in the UCLA activity scores, with the median score increasing from 2 prior to surgery to 5 at follow-up, Z = 1.890, *p* = 0.048, shown in Table 1.

**Figure 4:**
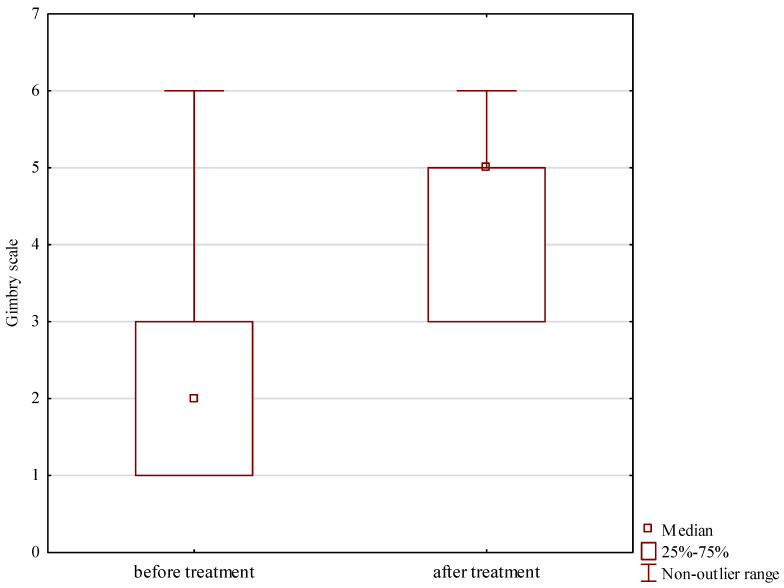
Grimby activity scores prior to and after treatment.

The Grimby activity score increased significantly from a median of 2 prior to surgery to 5 at long-term follow-up, Z = 2.267, *p* = 0.023, shown in Table 1 and Figure 4.

**Figure 5:**
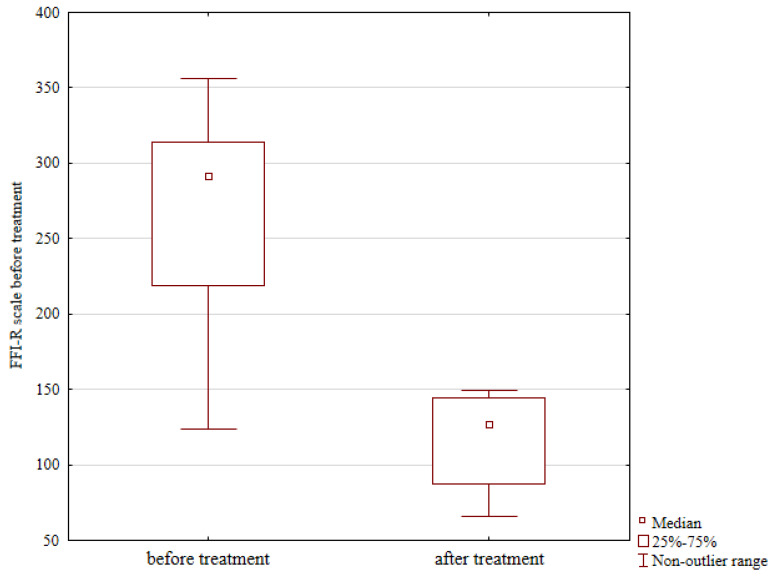
Functional outcomes prior to and after treatment, expressed as revised foot functional index (FFI-R) questionnaire scores.

The level of physical activity assessed with VAS also showed improvement, from a median preoperative score of 3 to 6 at follow-up; however, this difference was not statistically significant, Z = 0.353, *p* = 0.723, shown in Table 1. Functional outcomes based on FFI-R questionnaires showed a considerable improvement, from a median score of 292 points prior to surgery to 127 points at follow-up, Z = 0.244, *p* = 0.013, shown in Figure 5 and Table 1.

**Figure 6:**
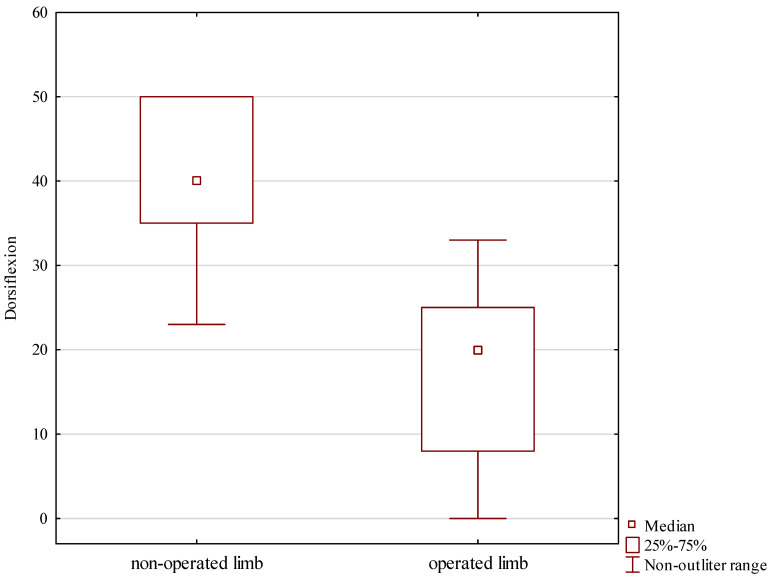
Foot dorsiflexion in the treated and intact limb.

Post-treatment follow-up measurements revealed a median dorsiflexion at the treated ankle joint of 20 degrees, whereas that at the intact ankle was 40 degrees, Z = 2.666, *p* = 0.007, shown in Table 2 and Figure 6.

**Figure 7:**
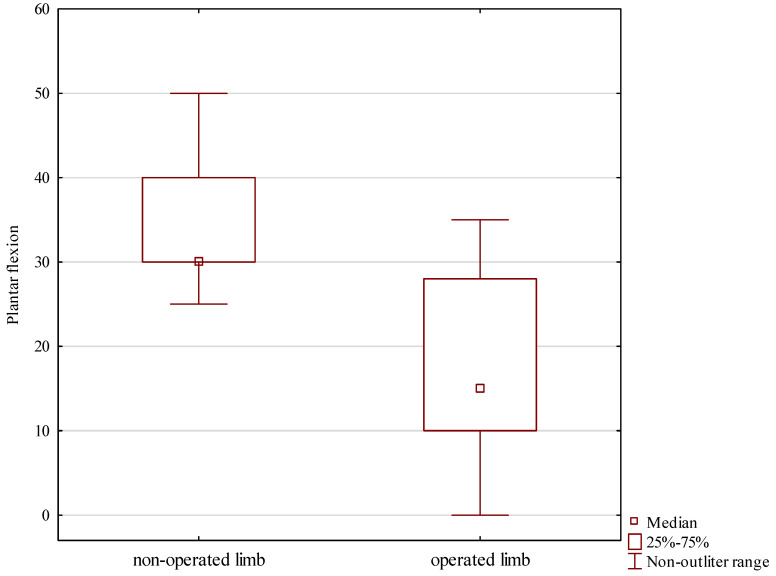
Foot plantar flexion in the treated and intact limb.

Plantar flexion showed a similar asymmetry, with a median 15 degrees at the treated ankle and 30 degrees at the intact ankle, Z = 1.874, *p* = 0.007, shown in Table 2 and Figure 7.

The median range of inversion at the ankle joint was 5 degrees in the treated limb and 15 degrees in the intact limb, with the difference being statistically significant, Z = 1.741, *p* = 0.007, shown in Table 2. The median range of eversion also showed a difference, with a median 8 degrees in the treated limb and 15 degrees in the intact limb; however, this difference was not statistically significant, Z = 0.325, *p* = 0.683, shown in Table 2.

## 4. Discussion

The aim of our study was to assess ankle functionality, the level of physical activity, and the range of motion at the ankle joint in patients with calcaneal fractures treated surgically with a Polish modification of the Ilizarov method. Following treatment, we observed a considerable improvement in the levels of activity measured via the UCLA and Grimby activity scores. There was also a significant post-treatment functional improvement measured with an FFI-R questionnaire. Our study showed that the extent of foot dorsiflexion, plantar flexion, and inversion at the ankle joint in the treated limb were lower than those in the intact limb. These results partly support our research hypothesis.

According to Roaas, the normal range of motion at the ankle ranges from 5 to 40 degrees for dorsiflexion, from 10 to 55 degrees for plantar flexion, and from 15 to 50 degrees for inversion and eversion [30]. Intra-articular calcaneal fractures continue to pose a challenge for orthopedic surgeons due to the structural complexity of the ankle joint and a high risk of postoperative complications [5,7,8,11,12,13]. Some authors suggested that restoring the normal structure of the foot may be one of the conditions for restoring normal ankle joint function [5,6,11,12]. On the other hand, some authors reported good functional outcomes without achieving the normal shape of the calcaneus and foot or achieving poor functional outcomes despite successful calcaneal fracture reduction [6,11,13]. Impaired function and mobility at the ankle joint often hinders a return to full performance and may be one of the causes of permanent disability [2,3,6,8]. Therefore, we recommend early rehabilitation with exercises aimed at increasing the range of motion and walking, which accelerates the return to everyday activities and yields more satisfactory outcomes [5,8,18].

There have been no studies assessing the level of physical activity after calcaneal fracture treatment. Some authors assessed the level of physical activity following ankle joint arthrodesis with the Ilizarov method [31] and following derotation corticotomy procedures with the Ilizarov method [32]. They observed good physical activity outcomes after ankle joint arthrodesis with an Ilizarov fixator and after derotation corticotomy procedures with the Ilizarov method [31,32]. Our study showed a significant improvement in UCLA and Grimby activity scores after treatment, which is a good outcome and is similar to those reported in the literature [31,32]. Improved activity scores following treatment with the Ilizarov method may have been related to the patients’ ability to ambulate with weight bearing very soon after treatment and with the minimally invasive nature of the surgery [3,5,8].

Schepers et al. assessed 14 patients after open reduction internal fixation and 1 patient after arthrodesis in patients with displaced calcaneal fractures [14]. The median FFI was 18 (interquartile range 6–37), which indicates good activity outcomes after surgery [14]. In another study, 90% of patients after calcaneal fracture treatment were able to resume work [19]. Long-term follow-up by Ibrahim demonstrated no differences between surgically and conservatively treated patients in terms of functional outcomes [20]. The mean FFI was 24.4 in patients treated conservatively and 26.9 in patients treated surgically [20]. Hashemi and his team conducted a retrospective study in which 60 patients with type II intra-articular calcaneal fracture according to the Sanders classification were assessed [21]. All patients underwent open reduction and internal fixation (ORIF) using a lateral approach [21]. Patients were divided into two groups: one with bone allograft and the other without allograft. The study results showed that the average foot function index (FFI) for the entire group of study patients was 9.1. For patients who had a bone allograft, the mean FFI was 9.9, while for those without an allograft it was 5.2. Despite these differences, the statistical analysis did not show significant differences between the groups [21]. In a retrospective study conducted on 416 patients, factors influencing foot function after ankle fracture were assessed [22]. The mean foot function index (FFI) score for all patients was 33.7 [22]. Audet et al.’s analysis showed that body mass index (BMI), smoking, complications, and additional injuries were significant independent predictors of higher FFI scores, indicating poorer foot function [22]. Mastracci and colleagues conducted a prospective study in which 21 patients with calcaneal fractures were evaluated [23]. All patients underwent calcaneal fixation via a sinotarsal approach and completed 12 months of follow-up. In the study group, the average total foot function index (FFI) score was 15 [23]. Furthermore, there was no significant correlation between radiological findings and the total FFI score. This means that although radiological results may indicate proper bone fusion and appropriate anatomy, they do not always translate into better foot function according to the FFI scale [23].

Our patients also noted improved functional scores after treatment, which is consistent with reports by other authors [14,19,20,21,22,23]. Improved general function scores after treatment may be due to increased levels of physical activity and indicate good treatment outcomes. The improvement in functional results after treatment in our patients may be related to the reduction in the incidence of infections and prolonged wound healing associated with the Polish modification of the Ilizarov method in the treatment of calcaneal fractures. Reducing the number of implants in the Polish modification may also reduce pain after treatment, compared to a larger number of implants introduced into the foot by other methods, which also allows for improved function of patients after treatment. The Polish modification of the Ilizarov method in the treatment of calcaneal fractures allows one to achieve functional results similar to those assessed by other authors [14,19,20,21,22,23,24,31,32].

After calcaneal fracture treatment, the mean range of ankle motion (dorsiflexion and plantar flexion) was 53 degrees, which constituted 88% of its normal value [19]. The mean range of talocalcaneal (i.e., subtalar) joint motion was 20 degrees, which constituted 67% of the normal value [19]. Park et al. evaluated 61 males and 17 females with unilateral calcaneal fracture and were surgically treated with open reduction internal fixation [18]. The authors observed no significant difference in dorsiflexion between the intact limb (16.9°) and the treated limb (16°). However, there were significant differences between the intact and treated limbs in terms of plantar flexion (39.5° vs. 35.3°, respectively) and inversion (50.5° vs. 34.8°, respectively) [18].

In this study, we observed a limited range of motion at the ankle joint following calcaneal fracture treatment with the Ilizarov method. Such limited range of motion may be a result of an insufficiently long or overly restricted rehabilitation regimen and the formation of connective tissue adhesions and scars. Persistent edema and muscle atrophy following surgical treatment of calcaneal fractures are also possible and may also limit joint mobility [7]. Patients who underwent treatment of calcaneal fracture are recommended to undergo a longer and more intense rehabilitation and have periodic follow-up visits to monitor any improvements in ankle mobility. Some of the other authors who evaluated calcaneal fracture treatment reported a limited range of motion in terms of some ankle joint movements following treatment [18,19]. The Polish modification of the Ilizarov method, despite the use of only one Kirschner wire inserted into the foot bones, was associated with limited movement of the ankle joint. The following factors may also have influenced the limited movement of the ankle joint after treatment of calcaneal fractures: concomitant soft tissue injuries after a calcaneal fracture, relatively long immobilization of the ankle joint after a calcaneal fracture, and the possible development of post-traumatic adhesions and fibrosis of soft tissues. Notably, despite limited ankle mobility following treatment in our patients, their level of physical activity and functional scores improved. This may be due to the fact that in order to improve the mobility of the ankle and foot after treatment, the patients started to do more sports and increased their overall physical activity levels.

One limitation of our study is the lack of preoperative assessments in patients with calcaneal fractures. This stems from the fact that such fractures are most often a result of high-energy trauma, which not only typically co-occurs with other injuries but is also impossible to predict in advance [1,2,3]. Other authors have conducted retrospective analyses of treatment outcomes for calcaneal fractures [3,4,7,8,12,14,18,20] and included patients with concomitant musculoskeletal injuries [5].

Another limitation of our study is the small sample size, which is due to the relatively low incidence of calcaneal fractures, which are most commonly due to traffic accidents and workplace injuries [1,2,3]. Moreover, some of our patients resided in places located far from our center, which prevented them from returning for follow-up visits. Nonetheless, other studies assessing calcaneal fracture treatment also included small study populations [3,4,8,12,14,16].

The strengths of our study are the use of homogeneous surgery and rehabilitation protocols and the fact that all procedures were conducted by the same orthopedic surgeon. In the future, we are planning to expand this study by using a larger group of patients and extending the follow-up period.

## 5. Conclusions

The treatment of calcaneal fractures with the use of a Polish modification of the Ilizarov method helps achieve satisfactory functional outcomes.

Patients with calcaneal fractures treated with the Ilizarov method are recommended to have a longer and more intensive rehabilitation.

The range of ankle motion in the treated limb was limited in comparison with that in the intact limb; however, this did not greatly affect the patients’ return to their earlier, pre-injury level of physical activity.

## Figures and Tables

**Table 1:** Detailed functional assessment of patients before and after surgery.

Analyzed Variable		Before Treatment	After Treatment	*p* *
	Value			
UCLA scale	Q1	1	4	0.048
	Median	2	5	
	Q3	7	6	
Gimbry scale	Q1	1	3	0.023
	Median	2	5	
	Q3	3	5	
VAS Activity Scale	Q1	0	5	0.723
	Median	3	6	
	Q3	8	8	
FFI-R scale	Q1	219	87	0.013
	Median	292	127	
	Q3	314	144	

* Wilcoxon signed-rank test; Q1, Q3—1st and 3rd quartile.

**Table 2:** Detailed range of motion of patients.

Analyzed Variable		Operated Limbt	Non-Operated Limb	*p* *
	Value			
Dorsiflexion [degree]	Q1	8	35	0.007
	Median	20	40	
	Q3	25	50	
Plantar flexion [degree]	Q1	10	30	0.007
	Median	15	30	
	Q3	28	40	
Inversion [degree]	Q1	5	5	0.039
	Median	5	15	
	Q3	10	20	
Eversion [degree]	Q1	5	5	0.683
	Median	8	15	
	Q3	10	20	

* Wilcoxon signed-rank test; Q1, Q3—1st and 3rd quartile.

## Data Availability

The data presented in this study are available on request from the corresponding author.
